# Dimethyl 3,3′-[(4-nitro­phen­yl)methyl­ene]bis­(1*H*-indole-2-carboxyl­ate) ethanol hemisolvate

**DOI:** 10.1107/S2414314621000572

**Published:** 2021-01-26

**Authors:** Yu-Long Li, Jin Zhou, Hong Jiang, Hong-Shun Sun, Rui-Zhe Li, Sha-Li Liu, Xu-Dong Zhang

**Affiliations:** a Targeted MRI Contrast Agents Laboratory of Jiangsu Province, Nanjing Polytechnic Institute, Nanjing 210048, People’s Republic of China; b College of Chemistry and Molecular Engineering, Nanjing Tech University, Nanjing 211816, People’s Republic of China; Zhejiang University (Yuquan Campus), China

**Keywords:** crystal structure, bis­indole, MRI, contrast agents

## Abstract

There are two main molecules in asymmetric unit of the title compound, C_27_H_21_N_3_O_6_·0.5C_2_H_5_OH. In both, the indole ring systems are approximately perpendicular to each other, at dihedral angles of 69.3 (5) and 82.8(4)°. In the crystal, mol­ecules are linked by N—H⋯O and O—H⋯O hydrogen bonds into a three-dimensional supra­molecular architecture. The solvent ethanol mol­ecule acts as a donor, forming an O—H⋯O hydrogen bond, reinforcing the structure.

## Abstract 2

There are two main molecules in the asymmetric unit of the title compound in which the two indole ring systems are approximately perpendicular to one another, at dihedral angles of 69.3 (5) and 82.8 (4)°.

## Graphical abstract

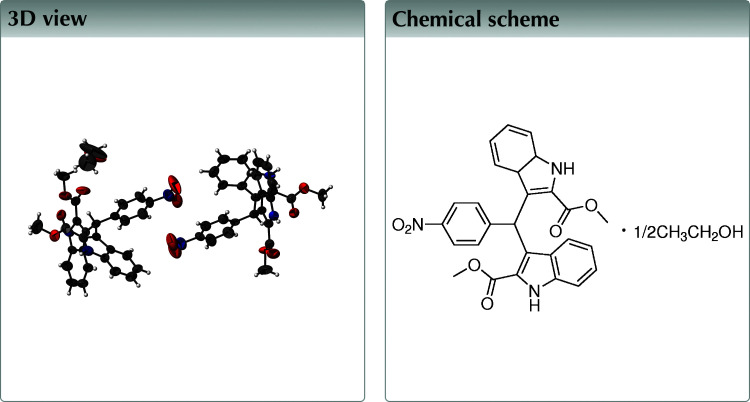

## Structure description

There are abundant bis­(indol­yl)methane derivatives in various terrestrial and marine natural resources (Sundberg, 1996 ▸). They can be used as precursors for MRI necrosis avid contrast agents (Ni, 2008 ▸). As part of our ongoing studies of bis­(indo­yl)methane compounds, we now report the synthesis and crystal structure of the title bis­(indoly)methane compound.

**Figure 1:**
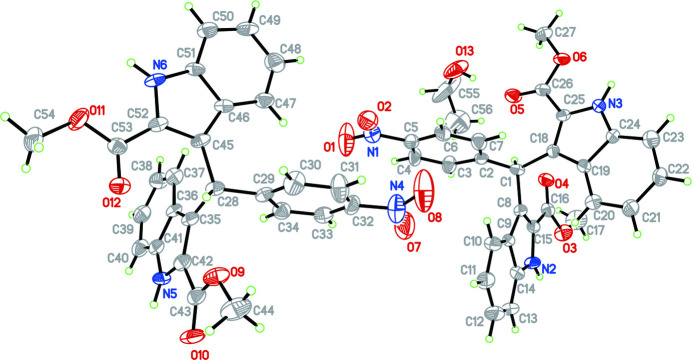
The mol­ecular structure of the title mol­ecule with the atom-labelling scheme. Displacement ellipsoids are drawn at the 30% probability level.

The mol­ecular structure of the title compound is shown in Fig. 1 ▸. In the first bisindole molhe two indole ring systems are nearly perpendicular to one another [dihedral angle = 69.3 (5)°] while the benzene ring (C2–C7) is twisted to the N2/C8–C15 and N3/C18–C25 indole ring systems by dihedral angles of 44.3 (3) and 77.6 (4)°, respectively. The carboxyl groups are approximately co-planar with the attached indole ring systems, the dihedral angles between the carboxyl groups and the mean plane of the N2/C8–C15 and N3/C18–C25 indole ring systems being 20.7 (4) and 3.8 (5)°, respectively. For the second bisindole molecule, the two indole ring systems are also nearly perpendicular to one another [dihedral angle = 82.8 (4)°] while the benzene ring (C29–C34) is twisted to the N5/C35–C42 and N6/C45–C52 indole ring systems with dihedral angles of 88.5 (5) and 81.8 (4)°, respectively.

**Figure 2:**
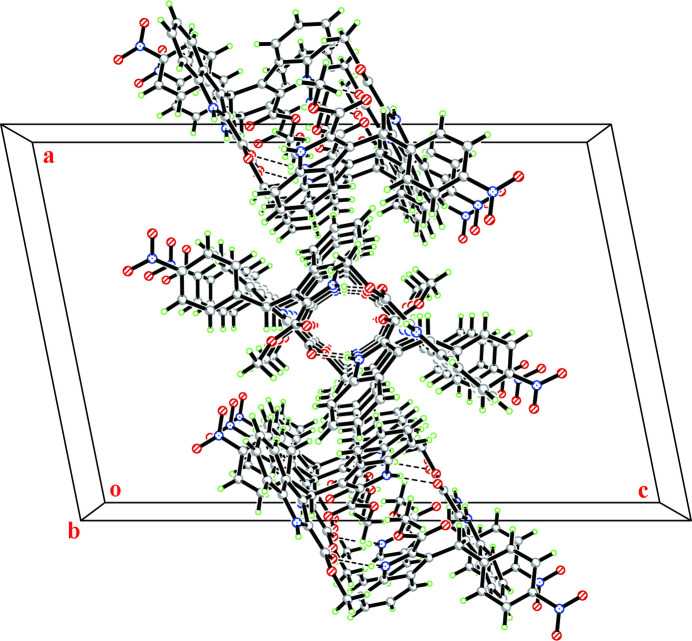
A packing diagram of the title compound. Hydrogen bonds are shown as dashed lines.

In the crystal, mol­ecules are linked by N—H⋯O and O—H⋯O hydrogen bonds into a three-dimensional supra­molecular architecture. The solvent ethanol mol­ecule acts as a donor, forming an O—H⋯O hydrogen bond, reinforcing the structure (Table 1 ▸, Fig. 2 ▸).

Several similar structures have been reported previously, *viz.* diethyl 3,3′-(phenyl­methyl­ene)bis­(1*H*-indole-2-carboxyl­ate) (Sun *et al.*, 2012 ▸), dimethyl 3,3′-[(3-nitro­phen­yl)methyl­ene]bis­(1*H*-indole-2-carboxyl­ate) ethanol monosolvate (Sun *et al.*, 2014 ▸), diethyl 3,3′-[(4-nitro­phen­yl)methyl­ene]bis­(1*H*-indole-2-carb­oxyl­ate) (Sun *et al.*, 2017 ▸) and diethyl 3,3′-[(3-fluoro­phen­yl)methyl­ene]bis­(1*H*-indole-2-carboxyl­ate) (Jiang *et al.*, 2020 ▸). In these structures, the indole ring systems are also nearly perpendicular to one another, making dihedral angles of 82.0 (5), 89.3 (5), 89.7 (5) and 88.3 (4)°, respectively.

## Synthesis and crystallization

Methyl indole-2-carboxyl­ate (1.75 g, 10 mmol) was dissolved in 20 ml of ethanol, and 4-nitro­benzaldehyde (0.76 g, 5 mmol) and concentrated HCl (0.5 ml) were added and the mixture was heated to reflux temperature for 2 h. After cooling, the white product was filtered off and washed thoroughly with ethanol. The reaction was monitored by TLC (AcOEt: hexane = 1:3). Yield 90%. Single crystals of the title compound suitable for X-ray analysis were obtained by slow evaporation of an ethanol solution.

## Refinement

Crystal data, data collection and structure refinement details are summarized in Table 2 ▸.

## Supplementary Material

Crystal structure: contains datablock(s) I. DOI: 10.1107/S2414314621000572/xu4043sup1.cif

Structure factors: contains datablock(s) I. DOI: 10.1107/S2414314621000572/xu4043Isup2.hkl

Supporting information file. DOI: 10.1107/S2414314621000572/xu4043Isup3.cml

CCDC reference: 2056440

Additional supporting information:  crystallographic information; 3D view; checkCIF report

## Figures and Tables

**Table 1:** Hydrogen-bond geometry (Å, °)

*D*—H⋯*A*	*D*—H	H⋯*A*	*D*⋯*A*	*D*—H⋯*A*
N2—H2*A*⋯O13^i^	0.86	2.00	2.814 (7)	157
N3—H3*A*⋯O4^ii^	0.86	2.05	2.892 (6)	166
N5—H5*A*⋯O12^iii^	0.86	2.01	2.848 (6)	163
N6—H6*B*⋯O10^iv^	0.86	2.16	2.984 (6)	160
O13—H13*B*⋯O5	0.82	1.91	2.733 (7)	177

Symmetry codes: (i) 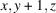 ; (ii) 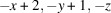 ; (iii) 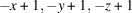 ; (iv) 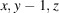 .

**Table 2:** Experimental details

Crystal data	
Chemical formula	2C_27_H_21_N_3_O_6_·C_2_H_6_O
*M*_r_	1013.00
Crystal system, space group	Monoclinic, *P*2_1_/*c*
Temperature (K)	293
*a*, *b*, *c* (Å)	18.248 (4), 10.304 (2), 27.541 (6)
β (°)	101.41 (3)
*V* (Å^3^)	5076.1 (18)
*Z*	4
Radiation type	Mo *K*α
μ (mm^−1^)	0.10
Crystal size (mm)	0.20 × 0.20 × 0.10

Data collection	
Diffractometer	Enraf–Nonius CAD-4
Absorption correction	ψ scan (North *et al.*, 1968 ▸)
*T*_min_, *T*_max_	0.97, 0.98
No. of measured, independent and observed [*I* > 2σ(*I*)] reflections	9324, 9324, 4039
*R*_int_	0.160
(sin θ/λ)_max_ (Å^−1^)	0.603

Refinement	
*R*[*F* ^2^ > 2σ(*F* ^2^)], *wR*(*F* ^2^), *S*	0.104, 0.213, 1.20
No. of reflections	9324
No. of parameters	676
No. of restraints	2
H-atom treatment	H-atom parameters constrained
Δρ_max_, Δρ_min_ (e Å^−3^)	0.36, −0.28

Computer programs: *CAD-4 EXPRESS* (Enraf–Nonius, 1994 ▸), *XCAD4* (Harms & Wocadlo, 1995 ▸) and *SHELXTL* (Sheldrick, 2008 ▸).

## full crystallographic data

### Crystal data

**Table:**

2C_27_H_21_N_3_O_6_·C_2_H_6_O	*F*(000) = 2120
*M**_r_* = 1013.00	*D*_x_ = 1.326 Mg m^−^^3^
Monoclinic, *P*2_1_/*c*	Mo *K*α radiation, λ = 0.71073 Å
*a* = 18.248 (4) Å	Cell parameters from 25 reflections
*b* = 10.304 (2) Å	θ = 9–12°
*c* = 27.541 (6) Å	µ = 0.10 mm^−^^1^
β = 101.41 (3)°	*T* = 293 K
*V* = 5076.1 (18) Å^3^	Block, colorless
*Z* = 4	0.20 × 0.20 × 0.10 mm

### Data collection

**Table:**

Enraf–Nonius CAD-4 diffractometer	9324 independent reflections
Radiation source: fine-focus sealed tube	4039 reflections with *I* > 2σ(*I*)
Detector resolution: 28.5714 pixels mm^-1^	*R*_int_ = 0.160
ω scans	θ_max_ = 25.4°, θ_min_ = 1.1°
Absorption correction: ψ scan (North *et al.*, 1968)	*h* = 0→22
*T*_min_ = 0.97, *T*_max_ = 0.98	*k* = 0→12
9324 measured reflections	*l* = −33→32

### Refinement

**Table:**

Refinement on *F*^2^	Primary atom site location: structure-invariant direct methods
Least-squares matrix: full	Secondary atom site location: difference Fourier map
*R*[*F*^2^ > 2σ(*F*^2^)] = 0.104	Hydrogen site location: inferred from neighbouring sites
*wR*(*F*^2^) = 0.213	H-atom parameters constrained
*S* = 1.20	*w* = 1/[σ^2^(*F*_o_^2^) + (0.050*P*)^2^ + 2.*P*] where *P* = (*F*_o_^2^ + 2*F*_c_^2^)/3
9324 reflections	(Δ/σ)_max_ < 0.001
676 parameters	Δρ_max_ = 0.36 e Å^−^^3^
2 restraints	Δρ_min_ = −0.28 e Å^−^^3^

### Special details

**Table:**

Geometry. All esds (except the esd in the dihedral angle between two l.s. planes) are estimated using the full covariance matrix. The cell esds are taken into account individually in the estimation of esds in distances, angles and torsion angles; correlations between esds in cell parameters are only used when they are defined by crystal symmetry. An approximate (isotropic) treatment of cell esds is used for estimating esds involving l.s. planes.
Refinement. Refinement of F^2^ against ALL reflections. The weighted R-factor wR and goodness of fit S are based on F^2^, conventional R-factors R are based on F, with F set to zero for negative F^2^. The threshold expression of F^2^ > 2sigma(F^2^) is used only for calculating R-factors(gt) etc. and is not relevant to the choice of reflections for refinement. R-factors based on F^2^ are statistically about twice as large as those based on F, and R- factors based on ALL data will be even larger.

### Fractional atomic coordinates and isotropic or equivalent isotropic displacement parameters (Å^2^)

**Table:**

	*x*	*y*	*z*	*U*_iso_*/*U*_eq_	
N2	0.9831 (3)	0.9393 (4)	0.14010 (17)	0.0539 (13)	
H2A	1.0127	1.0046	0.1411	0.065*	
C1	0.9320 (3)	0.5961 (5)	0.11280 (19)	0.0408 (14)	
H1A	0.9837	0.5646	0.1190	0.049*	
N1	0.7905 (4)	0.3205 (6)	0.2503 (2)	0.0752 (17)	
O1	0.8245 (4)	0.3088 (7)	0.2916 (2)	0.147 (3)	
C2	0.8937 (3)	0.5178 (5)	0.1471 (2)	0.0406 (13)	
O2	0.7277 (3)	0.2809 (5)	0.23639 (18)	0.0842 (15)	
O3	1.1030 (2)	0.8945 (4)	0.10153 (16)	0.0692 (13)	
N3	0.8863 (2)	0.4737 (4)	−0.01617 (16)	0.0488 (12)	
H3A	0.8903	0.4179	−0.0387	0.059*	
C3	0.9289 (3)	0.5036 (6)	0.1954 (2)	0.0576 (16)	
H3B	0.9762	0.5396	0.2055	0.069*	
N4	0.6521 (4)	0.5750 (8)	0.2207 (3)	0.092 (2)	
O4	1.0854 (2)	0.6789 (4)	0.09867 (14)	0.0573 (11)	
C4	0.8975 (4)	0.4384 (6)	0.2296 (2)	0.0641 (18)	
H4A	0.9227	0.4296	0.2623	0.077*	
O5	0.9967 (2)	0.3403 (4)	0.09448 (16)	0.0741 (14)	
C5	0.8273 (4)	0.3867 (6)	0.2139 (2)	0.0556 (17)	
N5	0.5897 (2)	0.5494 (4)	0.49143 (17)	0.0487 (12)	
H5A	0.5850	0.6178	0.5085	0.058*	
O6	0.9745 (2)	0.2731 (3)	0.01570 (14)	0.0635 (12)	
C6	0.7899 (3)	0.3955 (5)	0.1665 (2)	0.0551 (17)	
H6A	0.7425	0.3595	0.1567	0.066*	
N6	0.5217 (3)	−0.0105 (4)	0.39919 (18)	0.0523 (13)	
H6B	0.5035	−0.0759	0.4122	0.063*	
O7	0.7097 (4)	0.6377 (6)	0.2306 (2)	0.124 (2)	
C7	0.8245 (3)	0.4602 (6)	0.1328 (2)	0.0568 (16)	
H7A	0.8004	0.4647	0.0998	0.068*	
O8	0.6225 (3)	0.5476 (8)	0.1784 (2)	0.158 (3)	
C8	0.9376 (3)	0.7386 (5)	0.12664 (18)	0.0400 (13)	
C9	0.8841 (3)	0.8163 (5)	0.14583 (18)	0.0410 (14)	
O9	0.4550 (2)	0.5844 (4)	0.37946 (17)	0.0687 (13)	
O10	0.4921 (2)	0.7300 (4)	0.43938 (17)	0.0739 (13)	
C10	0.8120 (3)	0.7974 (6)	0.1546 (2)	0.0534 (16)	
H10A	0.7893	0.7165	0.1491	0.064*	
C11	0.7748 (3)	0.8978 (6)	0.1714 (2)	0.0627 (18)	
H11A	0.7269	0.8847	0.1773	0.075*	
O11	0.4208 (2)	0.0506 (4)	0.45411 (17)	0.0748 (14)	
C12	0.8081 (4)	1.0198 (6)	0.1798 (2)	0.071 (2)	
H12A	0.7821	1.0868	0.1915	0.085*	
O12	0.4222 (2)	0.2619 (4)	0.43498 (14)	0.0587 (11)	
C13	0.8771 (4)	1.0426 (6)	0.1712 (2)	0.0601 (18)	
H13A	0.8990	1.1240	0.1771	0.072*	
O13	1.0623 (4)	0.1713 (5)	0.1666 (2)	0.146 (3)	
H13B	1.0436	0.2240	0.1454	0.219*	
C14	0.9146 (3)	0.9412 (5)	0.1535 (2)	0.0476 (15)	
C15	0.9967 (3)	0.8174 (5)	0.12499 (19)	0.0434 (14)	
C16	1.0642 (3)	0.7862 (6)	0.1073 (2)	0.0462 (15)	
C17	1.1726 (3)	0.8759 (7)	0.0856 (3)	0.096 (3)	
H17A	1.1956	0.9586	0.0827	0.144*	
H17B	1.2053	0.8236	0.1094	0.144*	
H17C	1.1635	0.8331	0.0540	0.144*	
C18	0.9023 (3)	0.5722 (5)	0.05790 (18)	0.0379 (13)	
C19	0.8530 (3)	0.6490 (5)	0.02260 (19)	0.0415 (14)	
C20	0.8157 (3)	0.7670 (5)	0.0226 (2)	0.0546 (16)	
H20A	0.8200	0.8140	0.0519	0.066*	
C21	0.7729 (3)	0.8144 (6)	−0.0200 (2)	0.0617 (18)	
H21A	0.7488	0.8937	−0.0194	0.074*	
C22	0.7647 (3)	0.7462 (6)	−0.0643 (2)	0.0653 (18)	
H22A	0.7348	0.7804	−0.0927	0.078*	
C23	0.8000 (3)	0.6288 (6)	−0.0669 (2)	0.0634 (18)	
H23A	0.7940	0.5819	−0.0963	0.076*	
C24	0.8455 (3)	0.5837 (5)	−0.0231 (2)	0.0448 (14)	
C25	0.9202 (3)	0.4655 (5)	0.0326 (2)	0.0436 (14)	
C26	0.9679 (3)	0.3557 (5)	0.0517 (2)	0.0478 (15)	
C27	1.0198 (4)	0.1602 (5)	0.0313 (2)	0.076 (2)	
H27A	1.0215	0.1064	0.0031	0.115*	
H27B	1.0696	0.1870	0.0462	0.115*	
H27C	0.9985	0.1121	0.0550	0.115*	
C28	0.5378 (3)	0.3441 (5)	0.3791 (2)	0.0435 (14)	
H28A	0.4841	0.3632	0.3725	0.052*	
C29	0.5678 (3)	0.4072 (5)	0.3375 (2)	0.0425 (14)	
C30	0.5284 (3)	0.4046 (6)	0.2900 (2)	0.0698 (19)	
H30A	0.4821	0.3636	0.2835	0.084*	
C31	0.5543 (4)	0.4603 (8)	0.2512 (2)	0.081 (2)	
H31A	0.5262	0.4576	0.2191	0.097*	
C32	0.6227 (4)	0.5199 (6)	0.2613 (2)	0.0579 (17)	
C33	0.6644 (3)	0.5235 (5)	0.3077 (2)	0.0572 (17)	
H33A	0.7113	0.5624	0.3138	0.069*	
C34	0.6364 (3)	0.4690 (5)	0.3455 (2)	0.0550 (16)	
H34A	0.6644	0.4736	0.3776	0.066*	
C35	0.5697 (3)	0.4046 (5)	0.4289 (2)	0.0424 (14)	
C36	0.6299 (3)	0.3608 (5)	0.4667 (2)	0.0402 (13)	
C37	0.6785 (3)	0.2536 (6)	0.4714 (2)	0.0560 (16)	
H37A	0.6731	0.1910	0.4467	0.067*	
C38	0.7334 (3)	0.2426 (6)	0.5125 (2)	0.0635 (17)	
H38A	0.7663	0.1730	0.5148	0.076*	
C39	0.7419 (3)	0.3308 (6)	0.5509 (2)	0.0626 (18)	
H39A	0.7792	0.3182	0.5789	0.075*	
C40	0.6954 (3)	0.4379 (6)	0.5482 (2)	0.0607 (17)	
H40A	0.7010	0.4982	0.5738	0.073*	
C41	0.6397 (3)	0.4520 (5)	0.5056 (2)	0.0483 (15)	
C42	0.5479 (3)	0.5207 (5)	0.4455 (2)	0.0465 (14)	
C43	0.4962 (3)	0.6238 (6)	0.4224 (2)	0.0506 (15)	
C44	0.4048 (4)	0.6789 (6)	0.3526 (3)	0.089 (2)	
H44A	0.3779	0.6409	0.3224	0.134*	
H44B	0.3701	0.7067	0.3725	0.134*	
H44C	0.4329	0.7522	0.3449	0.134*	
C45	0.5442 (3)	0.1959 (5)	0.3796 (2)	0.0434 (14)	
C46	0.5886 (3)	0.1132 (5)	0.3559 (2)	0.0469 (15)	
C47	0.6385 (3)	0.1274 (6)	0.3240 (2)	0.0561 (17)	
H47A	0.6513	0.2098	0.3147	0.067*	
C48	0.6686 (4)	0.0199 (7)	0.3062 (3)	0.073 (2)	
H48A	0.7019	0.0302	0.2849	0.088*	
C49	0.6505 (4)	−0.1041 (6)	0.3196 (2)	0.0636 (19)	
H49A	0.6721	−0.1752	0.3071	0.076*	
C50	0.6018 (3)	−0.1247 (6)	0.3504 (2)	0.0596 (17)	
H50A	0.5888	−0.2081	0.3584	0.072*	
C51	0.5727 (3)	−0.0167 (5)	0.3692 (2)	0.0455 (15)	
C52	0.5040 (3)	0.1183 (5)	0.4053 (2)	0.0475 (15)	
C53	0.4451 (3)	0.1516 (6)	0.4333 (2)	0.0460 (15)	
C54	0.3624 (4)	0.0783 (6)	0.4808 (3)	0.084 (2)	
H54A	0.3478	−0.0005	0.4949	0.126*	
H54B	0.3804	0.1393	0.5068	0.126*	
H54C	0.3202	0.1144	0.4586	0.126*	
C55	1.1147 (7)	0.2243 (9)	0.1965 (4)	0.226 (8)	
H55A	1.1595	0.1774	0.1930	0.271*	
H55B	1.1060	0.2029	0.2292	0.271*	
C56	1.1354 (5)	0.3581 (9)	0.1991 (4)	0.151 (4)	
H56A	1.1776	0.3708	0.2256	0.227*	
H56B	1.1484	0.3838	0.1683	0.227*	
H56C	1.0942	0.4096	0.2049	0.227*	

### Atomic displacement parameters (Å^2^)

**Table:**

	*U*^11^	*U*^22^	*U*^33^	*U*^12^	*U*^13^	*U*^23^
N2	0.070 (4)	0.031 (3)	0.059 (3)	−0.009 (3)	0.007 (3)	−0.009 (2)
C1	0.042 (3)	0.035 (3)	0.045 (3)	0.004 (3)	0.007 (3)	−0.010 (3)
N1	0.098 (5)	0.071 (4)	0.058 (4)	−0.008 (4)	0.018 (4)	−0.002 (3)
O1	0.152 (6)	0.222 (8)	0.063 (4)	−0.065 (5)	0.013 (4)	0.040 (5)
C2	0.048 (4)	0.030 (3)	0.041 (3)	0.000 (3)	0.004 (3)	−0.004 (3)
O2	0.097 (4)	0.074 (3)	0.091 (4)	−0.020 (3)	0.041 (3)	−0.009 (3)
O3	0.056 (3)	0.062 (3)	0.093 (4)	−0.017 (2)	0.024 (3)	0.003 (3)
N3	0.071 (3)	0.037 (3)	0.036 (3)	0.001 (3)	0.005 (2)	−0.002 (2)
C3	0.054 (4)	0.068 (4)	0.047 (4)	−0.012 (3)	0.001 (3)	0.007 (3)
N4	0.079 (5)	0.134 (6)	0.064 (4)	0.005 (4)	0.016 (4)	0.029 (5)
O4	0.063 (3)	0.051 (3)	0.062 (3)	−0.002 (2)	0.023 (2)	−0.011 (2)
C4	0.081 (5)	0.073 (4)	0.033 (4)	−0.014 (4)	−0.001 (3)	0.010 (3)
O5	0.107 (4)	0.050 (3)	0.054 (3)	0.028 (3)	−0.011 (3)	−0.005 (2)
C5	0.070 (5)	0.046 (4)	0.059 (5)	−0.006 (3)	0.033 (4)	−0.002 (3)
N5	0.061 (3)	0.028 (3)	0.057 (3)	−0.006 (2)	0.014 (3)	−0.011 (2)
O6	0.093 (3)	0.033 (2)	0.060 (3)	0.013 (2)	0.004 (2)	−0.008 (2)
C6	0.062 (4)	0.053 (4)	0.047 (4)	−0.018 (3)	0.005 (3)	−0.012 (3)
N6	0.062 (3)	0.021 (2)	0.072 (4)	0.001 (2)	0.009 (3)	0.000 (2)
O7	0.154 (6)	0.131 (5)	0.099 (5)	−0.043 (5)	0.055 (4)	0.009 (4)
C7	0.059 (4)	0.064 (4)	0.046 (4)	−0.008 (3)	0.005 (3)	−0.002 (3)
O8	0.111 (5)	0.280 (9)	0.076 (4)	−0.012 (5)	0.002 (4)	0.062 (5)
C8	0.047 (4)	0.032 (3)	0.041 (3)	−0.001 (3)	0.008 (3)	−0.001 (3)
C9	0.057 (4)	0.034 (3)	0.032 (3)	−0.001 (3)	0.008 (3)	−0.002 (3)
O9	0.067 (3)	0.043 (2)	0.089 (4)	0.019 (2)	−0.002 (3)	0.009 (2)
O10	0.094 (4)	0.028 (2)	0.100 (4)	0.004 (2)	0.020 (3)	−0.005 (2)
C10	0.056 (4)	0.044 (4)	0.061 (4)	−0.003 (3)	0.014 (3)	−0.011 (3)
C11	0.057 (4)	0.062 (4)	0.070 (5)	0.022 (4)	0.015 (4)	0.008 (4)
O11	0.075 (3)	0.049 (3)	0.109 (4)	−0.011 (2)	0.040 (3)	0.017 (3)
C12	0.079 (5)	0.054 (4)	0.084 (5)	0.019 (4)	0.025 (4)	−0.005 (4)
O12	0.069 (3)	0.040 (2)	0.072 (3)	0.004 (2)	0.025 (2)	−0.005 (2)
C13	0.074 (5)	0.036 (3)	0.071 (5)	0.009 (3)	0.017 (4)	−0.005 (3)
O13	0.187 (6)	0.069 (4)	0.136 (5)	−0.052 (4)	−0.079 (5)	0.024 (4)
C14	0.053 (4)	0.046 (4)	0.045 (4)	0.004 (3)	0.013 (3)	−0.005 (3)
C15	0.051 (4)	0.039 (3)	0.039 (3)	−0.009 (3)	0.006 (3)	−0.004 (3)
C16	0.041 (4)	0.053 (4)	0.042 (3)	−0.012 (3)	0.003 (3)	−0.007 (3)
C17	0.054 (5)	0.104 (6)	0.138 (7)	−0.024 (4)	0.037 (5)	−0.002 (5)
C18	0.039 (3)	0.039 (3)	0.036 (3)	−0.009 (3)	0.006 (3)	0.000 (3)
C19	0.049 (4)	0.038 (3)	0.038 (3)	−0.004 (3)	0.009 (3)	−0.005 (3)
C20	0.063 (4)	0.046 (4)	0.055 (4)	0.006 (3)	0.012 (3)	0.001 (3)
C21	0.071 (5)	0.058 (4)	0.054 (4)	0.019 (3)	0.009 (4)	0.004 (4)
C22	0.081 (5)	0.062 (4)	0.044 (4)	0.020 (4)	−0.010 (3)	0.009 (3)
C23	0.069 (4)	0.062 (4)	0.057 (4)	0.003 (4)	0.005 (3)	0.000 (4)
C24	0.056 (4)	0.038 (3)	0.040 (4)	0.005 (3)	0.009 (3)	0.004 (3)
C25	0.056 (4)	0.029 (3)	0.047 (4)	−0.003 (3)	0.014 (3)	−0.002 (3)
C26	0.053 (4)	0.036 (3)	0.056 (4)	0.001 (3)	0.015 (3)	−0.009 (3)
C27	0.109 (6)	0.042 (4)	0.079 (5)	0.021 (4)	0.021 (4)	0.007 (4)
C28	0.039 (3)	0.033 (3)	0.057 (4)	−0.003 (3)	0.005 (3)	−0.006 (3)
C29	0.039 (3)	0.030 (3)	0.057 (4)	0.004 (3)	0.005 (3)	0.005 (3)
C30	0.053 (4)	0.082 (5)	0.067 (5)	−0.018 (4)	−0.007 (4)	0.007 (4)
C31	0.066 (5)	0.128 (7)	0.043 (4)	−0.006 (5)	−0.002 (4)	0.015 (4)
C32	0.066 (5)	0.057 (4)	0.051 (4)	0.009 (4)	0.013 (4)	0.017 (3)
C33	0.059 (4)	0.047 (4)	0.064 (5)	−0.005 (3)	0.009 (4)	−0.005 (3)
C34	0.058 (4)	0.052 (4)	0.053 (4)	−0.004 (3)	0.005 (3)	−0.004 (3)
C35	0.039 (3)	0.028 (3)	0.061 (4)	−0.002 (3)	0.012 (3)	0.001 (3)
C36	0.044 (3)	0.026 (3)	0.050 (4)	−0.004 (3)	0.007 (3)	0.005 (3)
C37	0.051 (4)	0.055 (4)	0.059 (4)	−0.003 (3)	0.005 (3)	0.002 (3)
C38	0.070 (5)	0.056 (4)	0.059 (4)	0.006 (4)	−0.001 (4)	0.001 (4)
C39	0.065 (4)	0.057 (4)	0.062 (5)	−0.003 (4)	0.002 (3)	0.013 (4)
C40	0.075 (5)	0.055 (4)	0.049 (4)	−0.022 (4)	0.005 (3)	0.001 (3)
C41	0.057 (4)	0.037 (3)	0.052 (4)	−0.009 (3)	0.014 (3)	0.002 (3)
C42	0.040 (3)	0.041 (3)	0.057 (4)	−0.004 (3)	0.006 (3)	0.006 (3)
C43	0.045 (4)	0.042 (4)	0.068 (5)	−0.004 (3)	0.016 (3)	0.006 (3)
C44	0.071 (5)	0.060 (4)	0.129 (7)	0.013 (4)	0.000 (5)	0.013 (5)
C45	0.038 (3)	0.029 (3)	0.058 (4)	−0.009 (3)	−0.002 (3)	−0.002 (3)
C46	0.047 (4)	0.031 (3)	0.061 (4)	0.001 (3)	0.007 (3)	−0.011 (3)
C47	0.056 (4)	0.041 (4)	0.074 (5)	0.006 (3)	0.020 (4)	−0.005 (3)
C48	0.068 (5)	0.073 (5)	0.085 (5)	0.009 (4)	0.031 (4)	−0.009 (4)
C49	0.074 (5)	0.034 (4)	0.080 (5)	0.013 (3)	0.007 (4)	−0.010 (3)
C50	0.057 (4)	0.040 (4)	0.084 (5)	0.002 (3)	0.019 (4)	−0.005 (3)
C51	0.040 (3)	0.034 (3)	0.062 (4)	0.005 (3)	0.011 (3)	−0.013 (3)
C52	0.050 (4)	0.032 (3)	0.058 (4)	−0.006 (3)	0.004 (3)	−0.003 (3)
C53	0.042 (4)	0.045 (4)	0.049 (4)	−0.006 (3)	0.005 (3)	−0.011 (3)
C54	0.084 (5)	0.072 (5)	0.113 (6)	−0.009 (4)	0.060 (5)	0.010 (4)
C55	0.255 (14)	0.116 (9)	0.228 (14)	−0.099 (9)	−0.142 (11)	0.105 (9)
C56	0.115 (8)	0.165 (10)	0.157 (10)	−0.038 (7)	−0.013 (7)	−0.020 (8)

### Geometric parameters (Å, º)

**Table:**

N2—C15	1.362 (6)	C19—C24	1.410 (7)
N2—C14	1.371 (6)	C20—C21	1.364 (7)
N2—H2A	0.8600	C20—H20A	0.9300
C1—C8	1.516 (7)	C21—C22	1.391 (8)
C1—C2	1.515 (7)	C21—H21A	0.9300
C1—C18	1.522 (7)	C22—C23	1.380 (8)
C1—H1A	0.9800	C22—H22A	0.9300
N1—O1	1.189 (7)	C23—C24	1.402 (7)
N1—O2	1.205 (6)	C23—H23A	0.9300
N1—C5	1.479 (8)	C25—C26	1.461 (7)
C2—C3	1.366 (7)	C27—H27A	0.9600
C2—C7	1.380 (7)	C27—H27B	0.9600
O3—C16	1.349 (6)	C27—H27C	0.9600
O3—C17	1.436 (6)	C28—C29	1.509 (7)
N3—C24	1.349 (6)	C28—C35	1.517 (7)
N3—C25	1.365 (6)	C28—C45	1.531 (7)
N3—H3A	0.8600	C28—H28A	0.9800
C3—C4	1.372 (7)	C29—C30	1.364 (7)
C3—H3B	0.9300	C29—C34	1.383 (7)
N4—O8	1.217 (8)	C30—C31	1.376 (8)
N4—O7	1.217 (7)	C30—H30A	0.9300
N4—C32	1.449 (8)	C31—C32	1.368 (8)
O4—C16	1.211 (6)	C31—H31A	0.9300
C4—C5	1.375 (7)	C32—C33	1.353 (8)
C4—H4A	0.9300	C33—C34	1.368 (7)
O5—C26	1.202 (6)	C33—H33A	0.9300
C5—C6	1.352 (8)	C34—H34A	0.9300
N5—C41	1.360 (6)	C35—C42	1.367 (7)
N5—C42	1.375 (6)	C35—C36	1.429 (7)
N5—H5A	0.8600	C36—C37	1.406 (7)
O6—C26	1.330 (6)	C36—C41	1.409 (7)
O6—C27	1.442 (6)	C37—C38	1.359 (7)
C6—C7	1.392 (7)	C37—H37A	0.9300
C6—H6A	0.9300	C38—C39	1.381 (8)
N6—C51	1.363 (6)	C38—H38A	0.9300
N6—C52	1.384 (6)	C39—C40	1.385 (8)
N6—H6B	0.8600	C39—H39A	0.9300
C7—H7A	0.9300	C40—C41	1.401 (7)
C8—C15	1.358 (7)	C40—H40A	0.9300
C8—C9	1.440 (7)	C42—C43	1.478 (7)
C9—C10	1.399 (7)	C44—H44A	0.9600
C9—C14	1.402 (7)	C44—H44B	0.9600
O9—C43	1.333 (7)	C44—H44C	0.9600
O9—C44	1.437 (6)	C45—C52	1.372 (7)
O10—C43	1.198 (6)	C45—C46	1.422 (7)
C10—C11	1.367 (7)	C46—C47	1.392 (7)
C10—H10A	0.9300	C46—C51	1.432 (7)
C11—C12	1.395 (8)	C47—C48	1.369 (8)
C11—H11A	0.9300	C47—H47A	0.9300
O11—C53	1.307 (6)	C48—C49	1.388 (8)
O11—C54	1.438 (6)	C48—H48A	0.9300
C12—C13	1.346 (8)	C49—C50	1.361 (8)
C12—H12A	0.9300	C49—H49A	0.9300
O12—C53	1.214 (6)	C50—C51	1.378 (7)
C13—C14	1.390 (7)	C50—H50A	0.9300
C13—H13A	0.9300	C52—C53	1.482 (7)
O13—C55	1.259 (9)	C54—H54A	0.9600
O13—H13B	0.8200	C54—H54B	0.9600
C15—C16	1.447 (7)	C54—H54C	0.9600
C17—H17A	0.9600	C55—C56	1.427 (8)
C17—H17B	0.9600	C55—H55A	0.9700
C17—H17C	0.9600	C55—H55B	0.9700
C18—C25	1.376 (7)	C56—H56A	0.9600
C18—C19	1.426 (7)	C56—H56B	0.9600
C19—C20	1.394 (7)	C56—H56C	0.9600
			
C15—N2—C14	109.1 (5)	O6—C27—H27B	109.5
C15—N2—H2A	125.5	H27A—C27—H27B	109.5
C14—N2—H2A	125.5	O6—C27—H27C	109.5
C8—C1—C2	112.0 (4)	H27A—C27—H27C	109.5
C8—C1—C18	113.5 (4)	H27B—C27—H27C	109.5
C2—C1—C18	114.6 (4)	C29—C28—C35	112.2 (4)
C8—C1—H1A	105.2	C29—C28—C45	113.6 (5)
C2—C1—H1A	105.2	C35—C28—C45	112.7 (5)
C18—C1—H1A	105.2	C29—C28—H28A	105.9
O1—N1—O2	123.5 (7)	C35—C28—H28A	105.9
O1—N1—C5	118.3 (7)	C45—C28—H28A	105.9
O2—N1—C5	118.2 (6)	C30—C29—C34	116.7 (6)
C3—C2—C7	117.4 (5)	C30—C29—C28	121.2 (5)
C3—C2—C1	118.6 (5)	C34—C29—C28	122.1 (5)
C7—C2—C1	124.0 (5)	C29—C30—C31	122.7 (6)
C16—O3—C17	116.3 (5)	C29—C30—H30A	118.6
C24—N3—C25	108.7 (5)	C31—C30—H30A	118.6
C24—N3—H3A	125.6	C32—C31—C30	117.9 (6)
C25—N3—H3A	125.6	C32—C31—H31A	121.0
C2—C3—C4	122.7 (6)	C30—C31—H31A	121.0
C2—C3—H3B	118.6	C33—C32—C31	121.7 (6)
C4—C3—H3B	118.6	C33—C32—N4	119.5 (7)
O8—N4—O7	122.5 (7)	C31—C32—N4	118.8 (6)
O8—N4—C32	119.0 (7)	C32—C33—C34	118.9 (6)
O7—N4—C32	118.1 (7)	C32—C33—H33A	120.6
C3—C4—C5	117.6 (6)	C34—C33—H33A	120.6
C3—C4—H4A	121.2	C33—C34—C29	122.1 (6)
C5—C4—H4A	121.2	C33—C34—H34A	118.9
C6—C5—C4	122.7 (6)	C29—C34—H34A	118.9
C6—C5—N1	118.3 (6)	C42—C35—C36	105.5 (5)
C4—C5—N1	119.1 (6)	C42—C35—C28	125.2 (5)
C41—N5—C42	108.9 (5)	C36—C35—C28	129.2 (5)
C41—N5—H5A	125.5	C37—C36—C41	118.1 (5)
C42—N5—H5A	125.5	C37—C36—C35	134.0 (5)
C26—O6—C27	115.2 (5)	C41—C36—C35	107.8 (5)
C5—C6—C7	117.8 (6)	C38—C37—C36	119.3 (6)
C5—C6—H6A	121.1	C38—C37—H37A	120.3
C7—C6—H6A	121.1	C36—C37—H37A	120.3
C51—N6—C52	108.8 (5)	C37—C38—C39	122.4 (6)
C51—N6—H6B	125.6	C37—C38—H38A	118.8
C52—N6—H6B	125.6	C39—C38—H38A	118.8
C2—C7—C6	121.7 (6)	C40—C39—C38	120.6 (6)
C2—C7—H7A	119.1	C40—C39—H39A	119.7
C6—C7—H7A	119.1	C38—C39—H39A	119.7
C15—C8—C9	106.3 (5)	C39—C40—C41	117.6 (6)
C15—C8—C1	125.8 (5)	C39—C40—H40A	121.2
C9—C8—C1	127.9 (5)	C41—C40—H40A	121.2
C10—C9—C14	117.4 (5)	N5—C41—C40	130.6 (6)
C10—C9—C8	135.7 (5)	N5—C41—C36	107.3 (5)
C14—C9—C8	106.7 (5)	C40—C41—C36	122.0 (6)
C43—O9—C44	116.1 (5)	C35—C42—N5	110.5 (5)
C11—C10—C9	120.0 (6)	C35—C42—C43	133.6 (6)
C11—C10—H10A	120.0	N5—C42—C43	115.6 (5)
C9—C10—H10A	120.0	O10—C43—O9	123.8 (6)
C10—C11—C12	120.6 (6)	O10—C43—C42	124.9 (6)
C10—C11—H11A	119.7	O9—C43—C42	111.2 (5)
C12—C11—H11A	119.7	O9—C44—H44A	109.5
C53—O11—C54	114.6 (5)	O9—C44—H44B	109.5
C13—C12—C11	121.3 (6)	H44A—C44—H44B	109.5
C13—C12—H12A	119.3	O9—C44—H44C	109.5
C11—C12—H12A	119.3	H44A—C44—H44C	109.5
C12—C13—C14	118.3 (6)	H44B—C44—H44C	109.5
C12—C13—H13A	120.9	C52—C45—C46	107.4 (5)
C14—C13—H13A	120.9	C52—C45—C28	122.6 (5)
C55—O13—H13B	109.5	C46—C45—C28	129.9 (5)
N2—C14—C13	130.1 (6)	C47—C46—C45	137.0 (5)
N2—C14—C9	107.6 (5)	C47—C46—C51	116.8 (5)
C13—C14—C9	122.3 (6)	C45—C46—C51	106.2 (5)
C8—C15—N2	110.3 (5)	C48—C47—C46	120.0 (6)
C8—C15—C16	127.8 (5)	C48—C47—H47A	120.0
N2—C15—C16	121.8 (5)	C46—C47—H47A	120.0
O4—C16—O3	122.5 (5)	C47—C48—C49	121.1 (6)
O4—C16—C15	126.5 (5)	C47—C48—H48A	119.5
O3—C16—C15	111.0 (5)	C49—C48—H48A	119.5
O3—C17—H17A	109.5	C50—C49—C48	121.9 (6)
O3—C17—H17B	109.5	C50—C49—H49A	119.1
H17A—C17—H17B	109.5	C48—C49—H49A	119.1
O3—C17—H17C	109.5	C49—C50—C51	117.2 (6)
H17A—C17—H17C	109.5	C49—C50—H50A	121.4
H17B—C17—H17C	109.5	C51—C50—H50A	121.4
C25—C18—C19	106.1 (5)	N6—C51—C50	128.8 (5)
C25—C18—C1	124.0 (5)	N6—C51—C46	108.0 (5)
C19—C18—C1	129.8 (5)	C50—C51—C46	123.1 (5)
C20—C19—C24	117.1 (5)	C45—C52—N6	109.6 (5)
C20—C19—C18	136.6 (5)	C45—C52—C53	130.5 (5)
C24—C19—C18	106.3 (5)	N6—C52—C53	119.7 (5)
C21—C20—C19	120.6 (6)	O12—C53—O11	125.5 (5)
C21—C20—H20A	119.7	O12—C53—C52	121.8 (5)
C19—C20—H20A	119.7	O11—C53—C52	112.8 (5)
C20—C21—C22	121.2 (6)	O11—C54—H54A	109.5
C20—C21—H21A	119.4	O11—C54—H54B	109.5
C22—C21—H21A	119.4	H54A—C54—H54B	109.5
C23—C22—C21	121.2 (6)	O11—C54—H54C	109.5
C23—C22—H22A	119.4	H54A—C54—H54C	109.5
C21—C22—H22A	119.4	H54B—C54—H54C	109.5
C22—C23—C24	116.7 (6)	O13—C55—C56	127.6 (9)
C22—C23—H23A	121.7	O13—C55—H55A	105.4
C24—C23—H23A	121.7	C56—C55—H55A	105.4
N3—C24—C23	128.0 (5)	O13—C55—H55B	105.4
N3—C24—C19	108.8 (5)	C56—C55—H55B	105.4
C23—C24—C19	123.2 (5)	H55A—C55—H55B	106.0
N3—C25—C18	110.1 (5)	C55—C56—H56A	109.5
N3—C25—C26	121.6 (5)	C55—C56—H56B	109.5
C18—C25—C26	128.3 (5)	H56A—C56—H56B	109.5
O5—C26—O6	124.0 (5)	C55—C56—H56C	109.5
O5—C26—C25	124.7 (5)	H56A—C56—H56C	109.5
O6—C26—C25	111.3 (5)	H56B—C56—H56C	109.5
O6—C27—H27A	109.5		
			
C8—C1—C2—C3	−67.2 (6)	C35—C28—C29—C30	−154.9 (5)
C18—C1—C2—C3	161.6 (5)	C45—C28—C29—C30	75.9 (7)
C8—C1—C2—C7	112.2 (6)	C35—C28—C29—C34	25.8 (7)
C18—C1—C2—C7	−19.0 (7)	C45—C28—C29—C34	−103.4 (6)
C7—C2—C3—C4	−1.8 (9)	C34—C29—C30—C31	−0.2 (10)
C1—C2—C3—C4	177.6 (5)	C28—C29—C30—C31	−179.5 (6)
C2—C3—C4—C5	−0.4 (9)	C29—C30—C31—C32	0.3 (11)
C3—C4—C5—C6	1.4 (9)	C30—C31—C32—C33	0.6 (10)
C3—C4—C5—N1	−177.5 (6)	C30—C31—C32—N4	177.8 (7)
O1—N1—C5—C6	177.4 (7)	O8—N4—C32—C33	164.3 (7)
O2—N1—C5—C6	−2.3 (9)	O7—N4—C32—C33	−9.2 (10)
O1—N1—C5—C4	−3.6 (10)	O8—N4—C32—C31	−12.9 (11)
O2—N1—C5—C4	176.7 (6)	O7—N4—C32—C31	173.5 (7)
C4—C5—C6—C7	−0.2 (9)	C31—C32—C33—C34	−1.7 (10)
N1—C5—C6—C7	178.8 (5)	N4—C32—C33—C34	−178.8 (6)
C3—C2—C7—C6	3.1 (8)	C32—C33—C34—C29	1.8 (9)
C1—C2—C7—C6	−176.3 (5)	C30—C29—C34—C33	−0.9 (8)
C5—C6—C7—C2	−2.2 (9)	C28—C29—C34—C33	178.4 (5)
C2—C1—C8—C15	141.4 (5)	C29—C28—C35—C42	77.0 (7)
C18—C1—C8—C15	−86.9 (6)	C45—C28—C35—C42	−153.3 (5)
C2—C1—C8—C9	−35.8 (7)	C29—C28—C35—C36	−98.6 (6)
C18—C1—C8—C9	95.9 (6)	C45—C28—C35—C36	31.1 (8)
C15—C8—C9—C10	176.7 (6)	C42—C35—C36—C37	−176.1 (6)
C1—C8—C9—C10	−5.7 (10)	C28—C35—C36—C37	0.2 (10)
C15—C8—C9—C14	1.5 (6)	C42—C35—C36—C41	2.2 (6)
C1—C8—C9—C14	179.2 (5)	C28—C35—C36—C41	178.5 (5)
C14—C9—C10—C11	−1.8 (8)	C41—C36—C37—C38	−0.9 (8)
C8—C9—C10—C11	−176.5 (6)	C35—C36—C37—C38	177.3 (6)
C9—C10—C11—C12	0.2 (9)	C36—C37—C38—C39	2.2 (9)
C10—C11—C12—C13	0.6 (10)	C37—C38—C39—C40	−2.1 (10)
C11—C12—C13—C14	0.4 (10)	C38—C39—C40—C41	0.5 (9)
C15—N2—C14—C13	179.8 (6)	C42—N5—C41—C40	178.0 (6)
C15—N2—C14—C9	−0.8 (6)	C42—N5—C41—C36	1.3 (6)
C12—C13—C14—N2	177.2 (6)	C39—C40—C41—N5	−175.5 (6)
C12—C13—C14—C9	−2.1 (9)	C39—C40—C41—C36	0.7 (8)
C10—C9—C14—N2	−176.6 (5)	C37—C36—C41—N5	176.5 (5)
C8—C9—C14—N2	−0.5 (6)	C35—C36—C41—N5	−2.1 (6)
C10—C9—C14—C13	2.8 (8)	C37—C36—C41—C40	−0.6 (8)
C8—C9—C14—C13	179.0 (5)	C35—C36—C41—C40	−179.2 (5)
C9—C8—C15—N2	−2.1 (6)	C36—C35—C42—N5	−1.4 (6)
C1—C8—C15—N2	−179.8 (5)	C28—C35—C42—N5	−177.9 (5)
C9—C8—C15—C16	−178.3 (5)	C36—C35—C42—C43	170.9 (6)
C1—C8—C15—C16	4.0 (9)	C28—C35—C42—C43	−5.5 (9)
C14—N2—C15—C8	1.8 (6)	C41—N5—C42—C35	0.1 (6)
C14—N2—C15—C16	178.3 (5)	C41—N5—C42—C43	−173.8 (4)
C17—O3—C16—O4	−1.4 (8)	C44—O9—C43—O10	1.7 (9)
C17—O3—C16—C15	178.1 (5)	C44—O9—C43—C42	−177.4 (5)
C8—C15—C16—O4	−13.5 (9)	C35—C42—C43—O10	−167.3 (6)
N2—C15—C16—O4	170.7 (6)	N5—C42—C43—O10	4.7 (8)
C8—C15—C16—O3	167.0 (5)	C35—C42—C43—O9	11.8 (9)
N2—C15—C16—O3	−8.8 (7)	N5—C42—C43—O9	−176.2 (5)
C8—C1—C18—C25	151.5 (5)	C29—C28—C45—C52	−162.7 (5)
C2—C1—C18—C25	−78.0 (6)	C35—C28—C45—C52	68.3 (7)
C8—C1—C18—C19	−28.3 (8)	C29—C28—C45—C46	17.1 (8)
C2—C1—C18—C19	102.1 (6)	C35—C28—C45—C46	−111.9 (7)
C25—C18—C19—C20	−178.6 (6)	C52—C45—C46—C47	177.4 (7)
C1—C18—C19—C20	1.3 (10)	C28—C45—C46—C47	−2.4 (11)
C25—C18—C19—C24	−1.4 (6)	C52—C45—C46—C51	−0.4 (6)
C1—C18—C19—C24	178.5 (5)	C28—C45—C46—C51	179.8 (5)
C24—C19—C20—C21	1.3 (8)	C45—C46—C47—C48	−176.4 (7)
C18—C19—C20—C21	178.2 (6)	C51—C46—C47—C48	1.2 (9)
C19—C20—C21—C22	0.6 (9)	C46—C47—C48—C49	−0.1 (10)
C20—C21—C22—C23	−0.5 (10)	C47—C48—C49—C50	0.4 (11)
C21—C22—C23—C24	−1.3 (9)	C48—C49—C50—C51	−1.9 (10)
C25—N3—C24—C23	−178.0 (6)	C52—N6—C51—C50	−174.8 (6)
C25—N3—C24—C19	−0.1 (6)	C52—N6—C51—C46	0.4 (6)
C22—C23—C24—N3	−179.1 (6)	C49—C50—C51—N6	177.6 (6)
C22—C23—C24—C19	3.3 (9)	C49—C50—C51—C46	3.1 (9)
C20—C19—C24—N3	178.7 (5)	C47—C46—C51—N6	−178.3 (5)
C18—C19—C24—N3	1.0 (6)	C45—C46—C51—N6	0.0 (6)
C20—C19—C24—C23	−3.3 (8)	C47—C46—C51—C50	−2.8 (9)
C18—C19—C24—C23	179.0 (5)	C45—C46—C51—C50	175.5 (5)
C24—N3—C25—C18	−0.8 (6)	C46—C45—C52—N6	0.6 (6)
C24—N3—C25—C26	179.4 (5)	C28—C45—C52—N6	−179.6 (5)
C19—C18—C25—N3	1.4 (6)	C46—C45—C52—C53	−174.8 (5)
C1—C18—C25—N3	−178.5 (4)	C28—C45—C52—C53	5.0 (10)
C19—C18—C25—C26	−178.9 (5)	C51—N6—C52—C45	−0.6 (6)
C1—C18—C25—C26	1.2 (9)	C51—N6—C52—C53	175.4 (5)
C27—O6—C26—O5	−0.1 (8)	C54—O11—C53—O12	−0.2 (9)
C27—O6—C26—C25	−179.1 (5)	C54—O11—C53—C52	−178.7 (5)
N3—C25—C26—O5	−176.8 (6)	C45—C52—C53—O12	3.1 (10)
C18—C25—C26—O5	3.5 (10)	N6—C52—C53—O12	−171.9 (5)
N3—C25—C26—O6	2.2 (7)	C45—C52—C53—O11	−178.3 (6)
C18—C25—C26—O6	−177.5 (5)	N6—C52—C53—O11	6.7 (7)

### Hydrogen-bond geometry (Å, º)

**Table:**

*D*—H···*A*	*D*—H	H···*A*	*D*···*A*	*D*—H···*A*
N2—H2*A*···O13^i^	0.86	2.00	2.814 (7)	157
N3—H3*A*···O4^ii^	0.86	2.05	2.892 (6)	166
N5—H5*A*···O12^iii^	0.86	2.01	2.848 (6)	163
N6—H6*B*···O10^iv^	0.86	2.16	2.984 (6)	160
O13—H13*B*···O5	0.82	1.91	2.733 (7)	177

Symmetry codes: (i) *x*, *y*+1, *z*; (ii) −*x*+2, −*y*+1, −*z*; (iii) −*x*+1, −*y*+1, −*z*+1; (iv) *x*, *y*−1, *z*.
